# What else is new about social media influencers? Uncovering their relation and content strategies, and the downsides of being famous

**DOI:** 10.3389/fpsyg.2024.1437384

**Published:** 2024-09-05

**Authors:** Chen Lou, Xuan Zhou

**Affiliations:** Wee Kim Wee School of Communication and Information, Nanyang Technological University, Singapore, Singapore

**Keywords:** social media influencers, parasocial relation, human brand, self-branding, digital labor, well-being, in-depth interviews

## Abstract

Prior research on social media influencers (SMIs) often examined questions such as their model of communication with followers, ethical concerns, motivations, and ways of gaining capital. How influencers curate intense and intimate relations and strategize their content creation, and how the influencer industry takes a toll on their physical and psychological wellbeing should be carefully addressed. To fill in this gap, we conducted in-depth interviews with 20 SMIs. The findings advance the literature on influencers and influencer advertising by explicating the ways through which influencers maintain intimate and engaged relations with followers, including *providing value*, *creating emotional bond*, *interacting and co-creating with followers*, and *disclosing personal life*. Second, this research identifies and theorizes four principles – *authenticity*, *topic sensitivity*, *fact-checking*, and *strategic sharing of privacy* – under which influencers strategize content creations in building human brands. Last, our findings add to the ongoing literature on digital labor by expounding the downsides of influencers being digital labor. This research contributes to the understudied aspect regarding influencers’ wellbeing and strategies employed in content creation and relation management in the current influencer literature.

## Introduction

Social media influencers (SMIs) are content generators who enjoy popularity among a sizable number of captive followers and who wield influence over followers’ consumption-related behaviors and other behaviors (e.g., pro-social behaviors, health-or diet-related behaviors) (e.g., Abidin, 2015; Hudders et al., 2021). Earlier bloggers, recent vloggers, YouTubers (Boerman and Van Reijmersdal, 2020), and “Instafamous” (Djafarova and Rushworth, 2017) are all considered SMIs. Extant literature on influencers and influencer advertising often explicates the effectiveness of influencer advertising (e.g., Lou and Yuan, 2019), perspectives from the industry practitioners and influencers pertaining to influencers’ impact (Brooks et al., 2021), the particulars of virtual influencers (Mrad et al., 2022), ethical issues involved in influencer advertising (Van Der Goot et al., 2021), among others. Pertaining to influencers’ perspectives, current research has explored their perceived ethical issues of the influencer industry (Borchers and Enke, 2022), the process through which influencers gain celebrity capital (Brooks et al., 2021), and motivations to become content creators (Törhönen et al., 2020). In particular, Abidin (2015) conducted field work, including participant observations in the influencer industry, personal interviews, and archival research into media coverage of influencers and influencers-generated content, to investigate how influencers interact and appropriate intimacies with followers.

Nonetheless, prior research on influencers often focused on questions such as their model of communication with followers, ethical concerns, motivations, way of gaining capital. Against this backdrop, how influencers curate intense and intimate relations and strategize and brand their content, and how the influencer industry takes a toll on their physical and psychological wellbeing can be further addressed. This research is to fill this gap by offering a holistic investigation pertaining to the three key issues in influencers’ lives – relations with followers, content creation, and wellbeing (Hudders and Lou, 2023). We draw on theories or concepts such as parasocial relation (i.e., the illusory and enduring relation between audience and media characters (Horton and Wohl, 1956)), human brand (i.e., human self as the subject of marketing efforts (Thomson, 2006)) and self-branding to theorize how influencers build relations and build human brands through content creation. We also argue that influencers are digital labors (Fuchs and Sevignani, 2013) whose contents are marketable commodities and can create profits, and further investigate the negative impact of the influencer industry on their physical and psychological wellbeing.

Theoretically, first, the findings of this research advance the literature on influencers and influencer advertising by explicating the ways through which influencers curate and maintain intimate and engaged relations with followers, including *providing value*, *creating emotional bond*, *interacting and co-creating with followers*, and *disclosing personal life*. Second, this research identifies and theorizes the four principles – *authenticity*, *topic sensitivity*, *fact-checking*, and *strategic sharing of privacy* – under which influencers strategize their content creations in the process of building human brands, which greatly contributes to the current literature explicating the marketing efficacy of influencers being human brands (e.g., Ki et al., 2020; Kim and Kim, 2022). Last, the current findings add to the ongoing literature on digital labor by expounding the downsides of influencers being digital labor (e.g., Abidin, 2017; Meisner and Ledbetter, 2022). Overall, this research sheds light on the understudied topics pertaining to influencers’ wellbeing and their content/relation strategies in the influencer literature.

## Literature review

### Social media influencers (SMIs)

Social media influencers (SMIs) refer to content generators who regularly share domain and/or entertaining knowledge and narrate their daily lives on one or multiple social media platforms and who thus have attracted a sizable number of followers (De Veirman et al., 2017). This phenomenon emerged earlier in blogs in 2015 (Borchers, 2019). More importantly, marketers and brands consider influencers to carry substantial marketing value and selling power and thus often employ them in sponsored campaigns promoting products and brands (Lou and Yuan, 2019). Recent research has explored numerous factors that explain the marketing efficacy of SMIs, including influencer credibility (expertise, attractiveness, trustworthiness, and similarity) and the value of influencer-generated content (Lou and Yuan, 2019), the influencer-follower relation (Boerman and Van Reijmersdal, 2020), influencer types (e.g., micro-versus mega-influencers) (Park et al., 2021), product divergence and the number of followers (De Veirman et al., 2017), among others. Specifically, Lou (2022) argues that the enhanced parasocial relation between influencers and followers, namely a trans-parasocial relation that is collectively reciprocal, interactive, and co-created by both influencers and followers, greatly accounts for the effectiveness of influencer advertising.

Besides exploring the marketing efficacy of influences, recent research has also looked into the role of SMIs in promoting prosocial behaviors, including fit influencers promoting healthy food (Folkvord et al., 2020), travel SMIs disseminating health-related messages during the pandemic (Femenia-Serra et al., 2022), and green influencers touting environmentally friendly products (Pittman and Annika, 2021) or sustainable consumption (Yıldırım, 2021). Despite the positive roles that SMIs play in shaping desirable behaviors, SMIs also exert negative implications on followers’ psychological and physical wellbeing (e.g., Chae, 2018; Pilgrim and Bohnet-Joschko, 2019; Smit et al., 2020). For instance, Chae (2018) conducted a two-wave online survey among South Korean women and found that their social media usage and personality traits (public self-consciousness and self-esteem) influence their envy toward influencers through social comparison with influencers. The frequency of watching influencers-generated vlogs also positively affects children’s consumption of unhealthy beverages (Smit et al., 2020).

Collectively, SMIs wield considerable influence over consumers’ consumption behaviors, health-related decision-making, and other behaviors (travel, fitness). We explore one of the explanatory factors – the parasocial relation or trans-parasocial relation between influencers and followers (Boerman and Van Reijmersdal, 2020; Lou, 2022) – that laregly accounts for this sweeping influence below.

### Parasocial relation and trans-parasocial relation

Horton and Wohl (1956) described the process of symbolic interaction between mass media audiences and performers as “parasocial interaction,” namely a “simulacrum of conversational give-and-take” (p. 215) that audience experience when being exposed to media personae or media characters. This parasocial interaction speaks to an illusory or imaginary intimate reciprocal social interaction with media characters perceived by mass media audiences, which often occurs or is confined to each single media exposure situation. Similarly, Hartmann and Goldhoorn (2011) concur that media users’ parasocial interaction is “characterized by a felt reciprocity with a TV performer that comprises a sense of mutual awareness, attention, and adjustment” (p. 1107). Parasocial relation, however, describes more than momentary illusory parasocial experiences felt by media users, but captures a more enduring and asymmetrical relation that media audience developed with media characters (Horton and Wohl, 1956). Specifically, media users or audiences often know a lot about the media performers/characters and develop an imaginary intimacy with them whereas the media performers/characters know little about media users/audience or rarely reciprocate.

Since the term parasocial relation was coined in the traditional era of TV and radio, it did not entail the two-way interactions that now are afforded by social media. Nevertheless, the assumptions of parasocial relation still hold when being applied to describe illusory one-sided, non-reciprocal relations developed among social media users (e.g., Colliander and Dahlén, 2011). However, pertaining to the relation between SMIs and followers, drawing on the perspectives of influencers, Abidin (2015) enumerates how this relation differs from parasocial relation in different ways. In particular, Abidin (2015) describes the influencer-follower relation to be contingent on intimacies, compared to parasocial relation developed based on the media characters’ theatrics. Second, she describes the influencer-follower relation to be co-constructed by both parties, whereas parasocial relation is often constructed by content producers of the media characters. Third, she argues that the influencer-follower relation is flat, bi-directional and interactive, whereas parasocial relation is often hierarchical, unidirectional, and non-interactive. Taking a step further, Lou (2022) theorized this new relation between influencers and followers as an enhanced format of parasocial relation, namely a trans-parasocial relation. She further explicates it as being collectively reciprocal, (a) synchronously interactive, and co-created. Specifically, SMIs often (1) reciprocate by proactively and regularly catering to followers’ collective or representative requests, comments, and inquiries, (2) (a)synchronously interact with followers via real-time live streaming or other means, and (3) co-created their content and interactions by actively soliciting followers’ input and suggestions (Lou, 2022).

While Lou (2022) systematically theorizes the influencer-follower relation from the followers’ perspective, we are interested in further investigating, from the influencers’ perspective, how they strategically develop and maintain their relations with followers. Thus, we ask:

**RQ1:** How and through which means do influencers strategize and develop their relations with followers?

Besides the role that the intimate trans-parasocial relation plays in enhancing followers’ loyalty and attachment to SMIs, SMIs, being human brands, also contribute to building a strong bond with followers and earning followers’ commitment to them via valuable content creations and brand building (Ki et al., 2020). We draw on the concept of human brand to explicate how SMIs curate their contents that help to forge this strong bond and wield influence over followers.

### Human brand, SMI’s self-branding, and content strategies

Brands in marketing often describe firms, products, and services in terms of perceived quality, image, and so on (Thomson, 2006). A human brand refers to “any well-known persona who is the subject of marketing communication efforts” and who possesses features and associations of a brand (Thomson, 2006: p. 104). Prior research has listed actors, musicians, politicians, athletes, CEOs, and idols as human brands and also demonstrated that human brands forge in-depth engagement with their audiences and earn their attachment (Thomson, 2006; Huang et al., 2015; Moulard et al., 2015). Specifically, Huang et al. (2015) examined and identified three factors – achievement vanity trait, variety seeking, and peer norms regarding idol worship – as the antecedents of idol attachment, which subsequently enhances human brand loyalty. The appeal of human brands in marketing is premised on the belief that the associations and traits that audiences perceive of human brands can transfer to promoted brands, products, and/or services via the use of a marketing mix (Williams et al., 2015). When human brands can meet audiences’ needs, such as autonomy (self-determination in choice and action), relatedness (closeness or intimacy), and competence, audiences tend to develop an intense bond with those human brands (e.g., Thomson, 2006; Ki et al., 2020).

Recently, literature on SMIs also describes SMIs as human brands, as SMIs cultivate their celebrity capital through curating valuable content and brandable personae on interactive social media (Ki et al., 2020; Kim and Kim, 2022). Kim and Kim (2022) argue that SMIs differ from those aforementioned traditional human brands by conducting two-way interactions with audiences. Indeed, SMIs build their brands by constantly curating and co-creating useful content with followers through interactive two-way communications (Lou, 2022), and such content has been found to bring value and satisfy followers’ needs (Lou and Yuan, 2019). In other words, SMIs’ content creation and curation are essentially the counterparts of the “theatrics” or “performance” of traditional human brands like celebrities or athletes, which help to convey their personalities to followers and contribute to followers’ attachment to them (Lou, 2022).

A few existing studies have explored SMI’s strategic self-branding practices on social media (e.g., Raun and Christensen-Strynø, 2022; Kim and McDonald-Liu, 2023; Miguel et al., 2024). For instance, Kim and McDonald-Liu (2023) argued that SMIs on Instagram established their personal brands through various self-presentation strategies, including self-promotion (e.g., bragging about oneself, disclosing luxury possessions), affiliation (e.g., showing fans in posted photos, using social hashtags), and authenticity (e.g., posting selfies without makeup or wearing casual clothes). Similarly, Miguel et al. (2024) suggested that foodie influencers (i.e., food lovers) on Instagram went through an audience-driven content creation process for self-branding, including content planning (referring to past experiences), media gathering (shooting at restaurants or events), editing (producing quality content), and publishing (posting content at planned intervals). On the other hand, Raun and Christensen-Strynø (2022) conducted a case study of two popular minority SMIs, Julie Vu (a trans-gender model) and Madeline Stuart (a model with Down syndrome). The authors described the self-branding strategy of minority SMIs as self-commercialization: garnering traffic through the capitalization of content and unique performance in front of the camera (Raun and Christensen-Strynø, 2022). Collectively, it is critical to examine how SMIs construct their content strategies to build human brands of themselves. We thus ask:

**RQ2:** What are influencers’ content strategies?

**RQ3:** How do influencers use those content strategies?

While visible SMIs function as human brands and promise marketing value to brands and marketers, most of them also often experience the “un(der)paid aspects of work in the social media industries” when performing constant creative labor in building self-branding (Meisner and Ledbetter, 2022: p. 1182). Content generators or creative labor like SMIs often struggle between empowerment and exploitation and experience harassment and discrimination in their work (Taylor, 2018; Meisner and Ledbetter, 2022). We thus further explore the life of influencers being digital labor and its downsides below.

### Influencers being digital labor and influencers’ well-being

Fuchs and Sevignani (2013) describe the concept of digital labor through the lens of Marx’s work and labor theory. They use digital labor to describe how contemporary corporate Internet platforms are based on the “exploitation of users’ unpaid labor,” and digital and social media users “engage in the creation of content and the use of blogs, social networking sites, wikis, microblogs, content sharing sites for fun and in these activities create value” (Fuchs and Sevignani, 2013: p. 237). In other words, digital and networked technologies users are digital labors, and user-generated-contents created by them are information commodities that can be advertised and used to create profits. Current literature on digital labor often investigates how social networking sites and digital technologies influence labor markets, work processes, and the boundaries of work (Neilson, 2018). As many contemporary online events and activities blur the line between work and play (Scholz, 2013), digital labors have been argued to face “precarious working conditions, the erosion of distinctions between work and leisure time, and the appropriation of unpaid and affective work” (Neilson, 2018: p. 538).

Recent research on digital labor often investigates working arrangements, contractual conditions, the unequal power, and vulnerability of digital labors in the so-called “creative industries” (e.g., content creators, SMIs) (e.g., Hayes and Silke, 2018; Neilson, 2018; Archer, 2019; Fieseler et al., 2019). For instance, Archer (2019) investigates how mom influencers navigate their roles as moms and market actors dealing with public relations practitioners, and how they handle precarity and vulnerability on their own. Hayes and Silke (2018) applied the concept of digital labor to study freelance journalists in the era of social media, and mainly focused on contractual conditions, work/life balance, and the differences in experiences between seasoned journalists and new entrants.

Relevant to SMIs, related concepts have been coined to investigate a particular segment of SMIs, including aesthetic labor describing fashion Instagram influencers (McFarlane and Samsioe, 2020) and sexualized labor explaining female Instagram influencers (Drenten et al., 2020). Aesthetic labor describes work in which “individuals are compensated, indirectly and directly, for their own body’s looks and affect” (Mears, 2014: p. 1332). McFarlane and Samsioe (2020) describe those fashion influencers as aesthetic labors and explore how they (aged 50 and above) redefine the expressions of cognitive age (feel, look, do, and interest age) through their content creations. Drenten et al. (2020) further define female Instagram SMIs conducting sexualized labors who monetize through expressing one’s sexiness. Sexualized labor herein refers to “an embodied performance that involves a complex, interrelated dynamic of emotion, aesthetics, and sexualization that cannot be separated from where it is placed” (Drenten et al., 2020: p. 45).

In particular, recent research has directed attention to influencers’ well-being in the digital economy (Levesque et al., 2023). As digital labors, influencers face the challenge of balancing their monetary value (e.g., paid sponsorship by brands) and authentic self-presentation online (Ashman et al., 2018; Levesque et al., 2023). For instance, through netnographic fieldwork, Ashman et al. (2018) concluded that young YouTubers’ quality of lives was negatively impacted by three pillars: the dynamics of competition (e.g., minimal start-up costs yet uncertain financial gain), the creativity dispositif (e.g., working hard and being innovative for success), and technologies of the self (e.g., conforming to a common criterion of beauty at the expense of diversity). Likewise, Levesque et al. (2023) examined the impact of engagement with COVID-19 topics on influencers’ well-being. During the hard times, influencers encountered self-presentation tensions that inhibited their well-being, including thwarted feelings of autonomy (potential risks associated with polarized issues) and authenticity-positivity tensions (influencers’ desire for authenticity and followers’ expectation for positivity) (Levesque et al., 2023). To address the tensions and enhance well-being, influencers adopted self-presentation solutions – fostering relatedness (e.g., interacting with followers) and negotiating competence (e.g., utilizing their influence to raise issue awareness) (Levesque et al., 2023).

Guided by prior research, we draw on the overarching concept of digital labor to further investigate how SMIs’ working arrangements and well-being are dictated by the unequal power, vulnerability, and laminarity of the creative influencer industry (Archer, 2019). Therefore, we ask:

**RQ4:** What is the negative impact of being an influencer on one’s physical and/or psychological well-being?

## Methods

We adopted a qualitative research method employing one-on-one in-depth interviews with influencers to address the proposed research questions. We chose in-depth interviews over other methods because (a) the rich and holistic data generated from in-depth interviews can help us to answer the mostly “what” and “how” questions asked in this research and place influencers in the right context (Tracy, 2013), (b) compared to focus group discussions, in-depth interviews with each individual influencer can alleviate the social desirability when some sensitive questions are discussed (e.g., personal struggles being an influencer, private topics that they will not share on social media), and (c) in-depth interviews help us to probe for further information when discussing a complex question such as content strategies or psychological wellbeing.

### Participants and procedure

We conducted in-depth interviews with 20 Singaporean SMIs to answer the research questions. We employed snowball sampling to recruit influencers and asked interviewees to refer us to other influencers whom they know. Meanwhile, we also tried to diversify our recruitment and enrolled influencers from different domains and those with varied numbers of followers. We received approval from the University’s Institutional Review Board (IRB) for this study and informed consent has been obtained from the interviewees. We stopped data collection when the data reached theoretical saturation, which means that the researchers “have enough data to build a comprehensive and convincing theory” (Morse, 1995: p. 148) and that the findings have sufficient conceptual rigor. Only those SMIs who are Singapore-based and who have experience with paid partnerships with brands were recruited. The enrolled SMIs aged between 19 and 44 years old (*M* = 27.6, *SD* = 6.89), including 11 females and nine males (see demographics in Table 1). Most of them are Chinese (*N* = 13, 65%), followed by 15% Malays (*N* = 3) and 10% Indians (*N* = 2). The duration of their years in the influencer industry ranged between 1 year and 14 years (*M* = 5.15, *SD* = 3.65). Their specialized domains span multiple areas, including lifestyle, beauty, fashion, food, travel, fitness, nutrition, as well as family and household. The most often-used social media sites by them consist of Instagram, TikTok, and YouTube. The number of their followers (on one of their dominant platforms) ranges between 5.7 K and 181.4 K. Most of them (*N* = 14, 70%) fall into the category of micro influencers (10 K – 100 K followers), along with three nano influencers (15%, 1 K – 10 K followers) and three macro influencers (15%, 100 K – 1 million followers).

**Table 1:** Demographics of interviewees.

	Pseudonym	Gender	Age	Ethnicity	No. of Years	Domain	Social media platforms used	No. of followers
1	Florentino	Male	19	Malay	3	Comedy, lifestyle, beauty	TikTok, Instagram, YouTube	99.7K^a^
2	Steven	Male	32	Chinese	7	Lifestyle	YouTube, Instagram, Twitch	27.2K^b^
3	Alicia	Female	22	Indian	9	Lifestyle, fashion	TikTok, Instagram	120.6K^a^
4	Rebecca	Female	44	Chinese	6	Travel, fashion, beauty, lifestyle	Instagram	32.3K^b^
5	Thomas	Male	35	Chinese	5	Lifestyle	TikTok, Instagram, Facebook	26.9K^b^
6	Johnny	Male	40	Chinese	14	Lifestyle, food, travel	Instagram, Facebook	31.6K^b^
7	Mark	Male	23	Chinese	1	Comedy	TikTok	181.4 K^a^
8	Amanda	Female	21	Chinese	6	Beauty, fashion, lifestyle	TikTok, Instagram	100.9K^a^
9	Cheryl	Female	25	Chinese	1	Lifestyle, beauty, fashion, fitness	TikTok, Instagram,	50.6K^a^
10	Melissa	Female	21	Chinese	3	Fashion, beauty	Instagram, YouTube, TikTok	53.7K^b^
11	Leon	Male	26	Chinese	3	Fitness	Instagram	90K^b^
12	Zelda	Female	30	Chinese	5	Lifestyle	Instagram	14K^b^
13	Kenneth	Male	32	Chinese	5	Lifestyle, travel	Instagram, Facebook	50.2K^b^
14	Ida	Female	23	Chinese	3	Lifestyle, fashion, beauty	Instagram	5.7K^b^
15	Bridget	Female	23	Caucasian	5	Lifestyle, fashion	Instagram	6.1K^b^
16	Lena	Female	23	Chinese	4	Lifestyle, fashion, beauty	Instagram	8.2K^b^
17	Teresa	Female	34	Anglo-Chinese	14	Lifestyle, fitness, beauty, nutrition, Fashion, family and household	Instagram, Facebook, Twitter, YouTube, TikTok	53.8K^b^
18	Daniel	Male	32	Malay	5	Lifestyle	Instagram, TikTok	25.2K^b^
19	Jonathon	Male	24	Indian	1	Lifestyle	Instagram, TikTok	20.5K^b^
20	Melissa	Female	23	Malay	3	Lifestyle, fashion, beauty	Instagram, TikTok	70.6K^b^

^a,bindicate the follower size on influencers’ dominant platform.^

^aindicates the number of followers on TikTok.^

^bindicates the number of followers on Instagram.^

Two trained research assistants followed an interview protocol guide (see Appendix A) and conducted the interviews independently. They both probed for further information when needed. All interviews were conducted in English over zoom. Each of the interviews was audio-recorded and transcribed verbatim by the two assistants. The interviews lasted around 40 min to 1.5 h.

### Data analysis

We followed a three-step protocol to code the transcripts. First, in the open-coding phase, two trained coders who were blind to the purpose of the study followed the constant comparative method – a qualitative analysis approach in grounded theory (Glaser and Strauss, 1968) – to code the transcripts. They independently read the transcripts line by line and assigned initial codes to the texts (e.g., codes such as “definition of SMIs,” “relationship with followers,” and “stressors”). Second, the two coders discussed the codes with the main researcher who has the theoretical knowledge and entered the axial/hierarchical-coding phase. The two coders and the researcher discussed all codes, identified, and excluded overlapping codes, and then grouped all the initial codes into second-level analytic themes (e.g., SMIs’ relation strategies, content strategies, and stressors) (Tracy, 2013). The existing literature and theories on parasocial/trans-parasocial relation, human brand, and digital labor guided the coders and researchers to categorize the codes. Lastly, the two coders and the main researcher discussed the second-level themes and reached a consensus on how they can be further combined or merged to answer each of the research questions (see Table 2). The main researcher further referred to the latest literature and theoretical arguments to theorize the new findings.

**Table 2:** Summary of the extracted themes, primary codes, and exemplary quotes.

Themes	Primary codes	Quotes
***Strategies for developing and maintaining relations with followers (RQ1)***		
Providing value	Provide a specific value	“Unless you are really providing a specific value for your audience, then the people are just going to find another influencer.”
	Put out good content	“I think just try to be positive and put out good content that they would enjoy.”
	Giveaways	“On some occasions, I do run some kind of like a giveaway to my followers. So to in order to so like, like, give back to them.”
	Promo code, benefit them	“Like um use my promo code. I mean, I do not earn anything lah, but if I think like it can benefit them, that would be good also lah.”
Creating emotional bond	Stay connected, respond to their concerns	“It’s just about staying connected, all about just responding to their concerns.”
	Sympathize, empathize	“I just want to sympathize and I want to empathize with you.”
	Encourage them	“I’ve had some followers who share maybe some of the struggles… I try my best to just encourage them.”
Interacting and co-creating with followers	Interact, live stream	“Q&A, and try to interact with them more, maybe live stream. I feel it’s a very good way to talk to them and know what they want from you.”
	Polls, ask me a question	“I will try and input those sticker like the polls, Like the ‘ask me a question’ kind of thing.”
	Talk, interview, chat	“Sometimes they can even talk with you – they can guest request, guest live. So they are like interviewing you or you can chat with them.”
	Engagement stickers	“I do use the engagement stickers from time to time, like voting, or ‘ask me’.
Disclosing personal life	Share what’s going on in my life	“I do more Instagram maybe through the form of stories, sharing what’s going on in my life from time to time.”
	Give an insight to my life	“I think give an insight to my life so that I do not feel like a robot to these people.”
	Where I am, what I’m eating	“I always post where I am and what I’m eating.”
	Post whatever I feel	“So I just post whatever I feel. If I’m out, if I’m playing tennis with my family, I’ll just post it. If I’m out bowling, I’ll just post it. Or if I’m going for coffee, I’ll just post it.”
***Content strategies of self-branding (RQ2)***		
Authenticity	Be genuine	“I think the most important guideline I give myself is always to be genuine.”
	Not go when I do not believe in	“I will not go and campaign for things that bring harm or I do not believe in.”
	Alignment with values	“I think it’s definitely that alignment with the values that you want to espouse, the kind of person you want to be.”
	Stay true to myself	“It’s important that I stay true to myself.”
	Find a balance	“So you have to really find a balance whereby we promote things that actually relates to us.”
Topic sensitivity	Not share politics & religion	“I will try not to share politics, I will try not to share religion.”
	Not share controversial stuff	“I do not really like to share controversial stuff or controversial topics like LGBT, or even like um politics.”
	Steer clar from touchy topics	“I steer clear from things that can be a very touchy topic.”
	Stay out of some topics	“On social media now, it’s a lot of drama, right? Especially in the local scene, and I stay out of that.”
Fact checking	Validation	“I think in social media, validation is something that we all need.”
	Face check	“When you have such a huge platform, you really have to fact check everything first and you know, not just like say whatever you think.”
Strategic sharing of privacy	Not to go in too deep	“I try not to go in too deep into these details as I like to keep my private life private also.”
	Not go in-depth	“I will not go in-depth about what goes on in our relationship.”
	Not share certain things	“I set out with the intention that I know there are certain things that I do not share about my personal life.”
	Not share bad stuff	“I would not exactly share any bad stuff that happens in my life because I prefer to deal with (it) privately.”
***Downsides of being digital labor (RQ3)***		
Unfair treatment by brands	Brands like to lowball	“Some brands do like to lowball us, I do not know why, but they refuse to accept that that is how much we offer.”
	Expect a lot but give you very little	“The only thing I find discomforting is when they expect a lot from you and they give you very little.”
	Do not value you	“I dislike it when they do not value you as an individual or the work that you do. So clients that take the piss in terms of deliverables.”
	Keep pressing you	“When they give you this certain requirement, and you are bounded by the contract, it does not feel good if they keep pressing you, your deadline, you need to fit this.”
	Do not give enough creative agency, too demanding	“I would really dislike if the client did not give me enough creative agency. Or if they are just too demanding and then you are not paying me enough for you to be so demanding.”
Followers’ hate and harassment	Comment toxic things	“There’s a lot less consequences for people to comment toxic things and all that.”
	Negative comments	“So if they hit you, then go like, well, your comments will be flooded with all the negative comments.”
	Hate comments	“There were times when I received hate comments from that, but I would delete them cos not nice mah then I just delete loh.”
The psychological toll of being an “influencer”	Maintain the level of aesthetics	“I have to so-called maintain the level of aesthetics for my post and the kind of partnerships that I’m working with because right now I’m doing fashion and beauty.”
	Live up to standards	“You’re not able to capture what they want, then you feel like oh shit, you feel like you are not able to live up to their standard and that’s quite pressurising sometimes.”
	Constantly think about content	“The need to think, constantly think about relatable content that draws people in.”
	Feel guilty	“So every time, I feel a post does not do well, I’ll feel damn guilty that they paid me so much money, and get only the amount of interest or likes, for example.”

## Results

The results show that most of the interviewees described themselves as content creators on social media who constantly share valuable content with followers and who influence the audiences’ emotions, motivations, and behaviors. The interviewees expressed that they gradually acquired a large group of followers by posting photos and filming videos of their daily lives. Soon after, brands and clients approached them for product endorsement and commercial partnerships. Most of the interviewees considered their relationship with followers as interactive, reciprocal, intimate, and emotionally connected.

Overall, we discerned that SMIs adopted a variety of strategies to develop and maintain relations with followers. Four themes emerged from the qualitative data, including (a) *providing value to followers*, (b) *creating emotional bond*, (c) *interacting and co-creating with followers*, and (d) *disclosing personal life*. Besides, SMIs meticulously craft their content to construct a recognizable brand identity online. We uncovered four principles of content creation for their self-branding, including (a) *authenticity*, (b) *topic sensitivity*, (c) *fact checking*, and (d) *strategic sharing of privacy*. However, our results also indicated how SMIs as digital labors in the industry negatively impacted their well-being. Three major themes emerged, including (a) *unfair treatment by brands*, (b) *followers’ hate and harassment*, and (c) *the psychological toll of being an “influencer.”* Below we elaborated on our findings based on the in-depth interviews.

### Strategies for developing and maintaining relations with followers (RQ1)

#### Providing value

Many interviewees highlighted the importance of offering concrete value (e.g., content value, monetary value) to followers to establish a favorable relationship. As Leon (a 26-year-old male) explained, “*There’re numerous influencers online. Unless you are really providing a specific value for your audience, people are just going to find another influencer*.” Since followers are often attracted by influencers’ content, many interviewees commit to offering value by creating relevant contents that resonates with followers. As Ida (a 23-year-old female) explained that, *“You must always try and identify what your followers like to see, what they engage with, and then cater to that.”*

Many interviewees also mentioned offering giveaways to provide monetary value to their followers. Giveaways, including free products or services (from sponsored brands), showcase influencers’ reciprocity and kindness to followers. For example, Amanda (a 21-year-old female) stated that:

*“Sometimes I will recommend to the brand, like, do you want to hold a giveaway instead of me receiving this extra product? So, there’s something for my followers to gain from it as well… It’s like they have a more rewarding experience also.”*

#### Creating emotional bond

Most interviewees emphasized that it is pivotal to connect with followers and create emotional bond with them, especially when followers shared personal concerns or struggles via comments or direct messages. For example, Jonathon (a 24-year-old male) mentioned he often caters to his followers when they need emotional support. Similarly, Florentino (a 19-year-old male) described how he encouraged one of his female followers with a relationship issue: “*…listen, you cannot force love, you know. If he does not want you, you walk away, know your own self-worth.”*

Daniel (an 18-year-old male) summarized the necessity of creating emotional bond with followers and cultivating intimacy:

*“It’s just about staying connected, all about just responding to their concerns. If they are sharing a particular issue, you just have to listen to them, pay attention to them… It’s always nice when they can rely on you to talk about certain things.”*

#### Interacting and co-creating with followers

Most interviewees indicated that they maintain frequent interactions with followers online and co-create content with followers. As Teresa (a 34-year-old female) shared, “*I would say that those that are in constant touch with me, those that make the effort to sort of reach out, ask questions, I’ve tried my best to respond to as many people as possible*.” Some interviewees also launched a “Q&A” or “ask me” session to directly engage followers. It is also noteworthy that many SMIs encourage followers to recommend topics and co-create content. Given the popularity of live streaming, many influencers create live sessions to talk to followers and interact with them synchronously. For example, Jonathon (a 24-year-old male) further explained:

*“On TikTok, there’s this feature called live, where you can go live and interact with the audience. They can ask you questions, just get to know you better also. These are some ways that really makes your personality shine forth and it connects with the audience better.”*

#### Disclosing personal life

Our interviewees also revealed that disclosing personal life (e.g., daily life, interesting happenings) is an effective approach to maintaining online presence, building rapport, and forming intimate relations with followers. For instance, Bridget (a 23-year-old female) explained that “*I just post whatever I feel. If I’m out, if I’m playing tennis with my family, If I’m out bowling, or if I’m going for coffee, I’ll just post it*.” Likewise, through these self-disclosing activities, Ida (a 23-year-old female) stated that “*I think giving an insight to my life so that I do not feel like a robot to these people.*” Teresa (a 34-year-old female) echoed this view and expected to establish a connection with followers by disclosing her personal life:

*“I feel like people are able to form a connection based on the experiences that I’m going through. If I am not feeling 100% confident about my body, I will post about that. Because people who are not 100% confident about themselves will go oh, I feel the same… it’s like you know me.”*

### Content strategies of self-branding (RQ2 and RQ3)

#### Authenticity

Most interviewees agreed to maintain a high level of authenticity in content creation and keep their personal brand as genuine as possible. In particular, as excessive partnerships with brands pose threat to authenticity, many interviewees were selective about sponsorships and only collaborated with brands that aligned with their values and personalities. For example, Ida (a 23-year-old female) shared that:

*“I will put out content or talk about things that I think will be useful for people, like I will not go and campaign for things that bring harm, or I do not believe in. I’ll be more careful of what I say because it reflects on my own personal branding.”*

Moreover, interviewees expressed that they try to strike a balance between advocating for brands and branding their authentic personalities. Amanda (a 21-year-old female) described:

*“By collaborating with all these brands, uh it really makes me question myself if my content is still genuine. I think that is something that a lot of creators struggle with, because if we over promote then it’s like we lose our own personality and people do not like that.”*

#### Topic sensitivity

Our interviewees were also sensitive about topic selection and have gut feelings regarding what topics may go viral and what may go awry. On the one hand, they mentioned that they often create the so-called “popular contents” that are expected to attract followers, such as unboxing videos, funny videos, aspirational content, and tutorials. For example, Mark (a 23-year-old male) stated that, “*We were doing more videos from what people were commenting and what they enjoyed seeing.*” On the other hand, interviewees are cautious with their sharing when it comes to sensitive or controversial topics, such as politics, religion, and LGBTQA-related issues. Several interviewees explained that they avoided such conversations because they hardly gain benefits from voicing out on these topics. As Melissa (a 23-year-old female) put, “*I stay out of those. I do not try to stir the pot. I do not try to cause any trouble*.” Kenneth (a 32-year-old male) agreed that influencers should not get involved in controversial topics as people with oppositive views will slam SMIs:

*“This kind of questions, there’s no right or wrong, right? It’s just how you look at it, so I try to avoid these topics. If not, when you step out, then people will bash you for their stand. It’s a never-ending game.”*

#### Fact checking

In order to protect their personal brands, many interviewees also considered it necessary to conduct fact checking to vet the contents before posting them. Given the prevalence of misinformation or fake news, Florentino (a 19-year-old male) noted that “*validation is something that we all need*.” Interviewees also explained that, as opinion leaders, they should only share opinions when they are 100% sure about the facts; otherwise, they are likely to mislead followers and hurt the credibility of their self-branding. Amanda (a 21-year-old female) shared how posting without fact checking causes trouble on social media:

*“Recently the Russian-Ukraine thing is going on, and then there was this donation page being held out… I straight away reposted it on my story and like 5 min later, I took it down, because people were responding: it’s not a valid platform to donate to… so you have to fact check all these things.”*

#### Strategic sharing of privacy

To maintain their brand identities, our interviewees also strategically shared their privacy and restricted the extent to which their private matters (e.g., families) could go public. For example, some interviewees were only willing to share positive private lives on social media. As Johnny (a 40-year-old male) explained:

*“For example, if like, let us say I have a dinner, my family, and then we have like, I post some photos of family and then like during mother’s day, I post about my mom’s. So I write about like, um, stuff. She, she done for me, that kind of thing. So it’s like something more positive.”*

Besides, most interviewees pointed out that there is a clear line for privacy protection, and they hold back from sharing private issues, such as family, relationships, and financial problems. Jonathon (a 24-year-old male) put that, “*it’s your personal life, keep it personal. I think when you blur that line, and you bring your personal life in there, things can get pretty messy*.” Some interviewees mentioned that the reason why they keep certain life aspects private is that they “*respect that there are other people and other parties involved*” (Daniel, a 32-year-old male). Kenneth (a 32-year-old male) echoed this view:

*“Let us say I went on a date with you, I would not want to show the world that I am on a date with you… So much of my information is already public, this is the one area I can keep private I would do it.”*

### Downsides of being digital labor (RQ4)

#### Unfair treatment by brands

Many interviewees shared the unpleasant experiences of working with abusive, demanding, and unreasonable brands or marketers. Mark (a 23-year-old male) complained that certain brands have “*unrealistic expectations and demands*.” Leon (a 26-year-old male) echoed this sentiment and elaborated how influencers work under this unbalanced power relation, “*you are bounded by the contract; it does not feel good if they keep pressing you, your deadline, you need to fit this, this picture does not fit within our requirements, you need to reshoot or retake*.” Moreover, some interviewees acknowledged that although they are trying hard to meet requirements, brands and clients tend to disrespect their work and underpay them, treating influencers as “*easy and cheap way of getting things done*” (Ida, a 23-year-old female). Melissa (a 23-year-old female) further shared that:

*“Some brands do like to lowball us, I do not know why, but they refuse to accept that that is how much we offer. They always, always, always try to lowball us. It’s very rare for brands to just accept how much you have quoted them.”*

#### Followers’ hate and harassment

While most of the time SMIs maintain harmonious and interactive relations with followers, our interviewees also indicated that followers could exert a negative impact on their well-being via harsh comments or harassment. Most interviewees mentioned that they encountered hatred or toxic comments from followers. For example, Melissa (a 21-year-old) emphasized that “*people can hide behind [screen], so they can comment anything they want; if they hit you, your comments will be flooded with all the negative comments*.” Mark (a 23-year-old male) encountered the same situation and commented on why toxic comments exist, “*there’s a lot less consequences for people to comment toxic things and all that.*”

Cheryl (a 25-year-old female) also described unsolicited and unexpected negative comments that coexist with positive comments from followers: *“…with good things there will be bad things, like there can be people who say like oh she looks so fake.”* Thomas (a 35-year-old male) echoed this sentiment:

*“The more attention people have on you, the more dirt they see on your shirt, you know what I mean? Especially that one stain… one stain of noodle stain, the bigger personality you are, the more people will realize, eh he got one noodle stain there, it happens what, it’s unavoidable.”*

Teresa (a 34-year-old female) recalled some negative comments that she received after some followers noticed that she gained weight, *“all your keyboard warriors will tell you that you look fatter than you used to or, you used to look better.”*

#### The psychological toll of being an “influencer”

Besides dealing with abusive brands or clients and the negative influence of followers, interviewees further revealed that their work performance and daily behaviors are constrained and regulated by their profession as an “influencer” (i.e., a person that needs to constantly influence others). Specifically, they described that being influencers means they are expected to constantly offer “high-quality,” “creative,” “the best,” and relevant content. Florentino (a 19-year-old male) described this pressure as, “*the need to think, constantly think about relatable content that draws people in, and more importantly, like sometimes our mental health, a bit too much for me and I need to take a break*.” Teresa (a 34-year-old female) held a similar viewpoint regarding maintaining relatability, “*a constant challenge and a bit of a stressor because you have got to remain on top of trends, and you have got to try and be relevant to as many people as possible*.” Further, interviewees mentioned that keeping up with the look as influencers also cause a lot of stress in their daily lives. Amanda (a 21-year-old female) described: *“Most of the times I’m actually dressed like super casual, just wear hoodie to work but on my Instagram, you see me dressing up and all that. I feel stressed like I’m not being who I am online.”*

## Discussion

The current findings extend the theoretical understanding of influencer-follower relation formation and maintenance, SMIs’ content strategies in the process of self-branding, as well as the downsides of being digital labor. These findings greatly contribute to the understudied aspects regarding influencers’ wellbeing and strategies employed in content creation and relation management in the current influencer literature. First, four themes that summarize how SMIs strategize and develop relations with followers emerge from the data, including (a) providing value, (b) creating emotional bond, (c) interacting and co-creating with followers, and (d) disclosing personal life. Second, we theorized four principles that guide SMIs’ content strategies in the process of self-branding: authenticity, topicality, fact-checking, and privacy. Last, we further explicate the downsides of being digital labor in the influencer industry and uncover three issues: (a) unfair treatment by brands, (b) followers’ hate and harassment, and (c) the psychological toll of being an influencer. We elaborate on the theoretical and practical implications below.

### Theoretical implications

First, we indeed found that SMIs either intentionally or unintentionally follow certain strategies to curate and maintain their intimate relations with followers. Corroborating with prior research on the characteristics of influencer-follower relation (e.g., Abidin, 2015; Lou, 2022), our interviewees also consider their relations with followers largely reciprocal, interactive, and co-created. Taking a step further, we shed light on the strategies that contribute to this so-called trans-parasocial relation (Lou, 2022) or interconnectedness (Abidin, 2015) between SMIs and followers. Specifically, these strategies – *providing value, creating emotional bond, interacting and co-creating with followers, and disclosing personal life* – correspond to the tenets of the trans-parasocial relation, an intimate and enhanced relation between influencers and fervid followers proposed by Lou (2022).

For instance, creating and providing value emerged as a top strategy advocated by many SMIs. This echoed the view of perceiving influencer-generated content as having informative value and entertainment value that satisfies followers’ needs (Lou and Kim, 2019; Lou and Yuan, 2019). Indeed, followers often pick SMIs to follow to fulfill their intrinsic needs and these fulfilled needs subsequently contribute to their attachment to SMIs (Ki et al., 2020). We also noticed that influencers attempt to provide monetary value via giving away sponsored products or services to followers. This signals SMIs’ altruistic intentions to create value for followers and SMIs expect to strengthen their bond with followers through such ways. Second, creating emotional bond counts toward another commonly used strategy by SMIs to develop relations with followers. We found that most SMIs engage in providing emotional support to followers and hope to increase followers’ attachment to them by doing so. Third, most SMIs mention that they constantly interact with their followers and invite content co-creation from followers. This finding again echoes the view of considering the influencer-follower relation as interactive and co-created (Abidin, 2015; Lou, 2022). SMIs believe that constant and regular interactions and co-creations with followers are effective and indispensable means to democratize their relations with followers and to strengthen the tie between them. Last, SMIs well recognize the importance of disclosing personal lives to followers to forge and enhance their relations with followers. This finding agrees with some recent research stressing how the depth and breadth of self-disclosure by SMIs shape followers’ trust in SMIs (Alrabiah et al., 2022). Disclosing personal lives and narratives that appear to be “genuine, raw and usually inaccessible aspects” (Abidin, 2015, p. 11) serves as an effective means to convey intimacies to followers.

The second theoretical contribution of this research lies in how it has theorized the principles – *authenticity*, *topic sensitivity*, *fact-checking*, and *strategic sharing of privacy* – that guide SMIs’ self-branding process. This advances the current literature explicating SMIs as human brands and their strategies in self-branding (e.g., Jun and Yi, 2020; Ki et al., 2020; Kim and Kim, 2022; Raun and Christensen-Strynø, 2022; Kim and McDonald-Liu, 2023; Miguel et al., 2024). Specifically, first, it is not surprising to see authenticity emerge as a top principle guiding SMIs’ self-branding. This echoes a recent finding arguing influencer authenticity (e.g., wearing casual clothing or posting selfies without makeup) helps to establish influencers’ personal brands (Kim and McDonald-Liu, 2023). We further add to this argument by showing that maintaining an authentic identity (e.g., be selective to sponsored deals) helps influencers to brand themselves better. Second, we found that SMIs often have seasoned experiences forecasting followers’ reactions to certain topics of their sharing. Well-versed SMIs know that certain topics can entice engagement whereas others may backfire. This self-taught knowledge or “instinct” regarding the relation between topic sensitivity and follower reactions helps them to navigate their self-branding practices. Third, we also found that SMIs are concerned with the veracity or credibility of their content sharing and often engage in fact-checking before posting social news or relevant content. Indeed, influencer credibility has been found to positively influence followers’ trust and parasocial relation with them (Lou and Kim, 2019; Lou and Yuan, 2019). SMIs, being content generators and opinion leaders, are thus expected to engage in gate-keeping activities before sharing to maintain their credibility. Last, SMIs also hold their privacy in high regard. While we know that SMIs constantly disclose their personal lives in exchange for following and brand sponsorships (Alrabiah et al., 2022), it may sound paradoxical to see that they also safeguard their privacy meticulously. Most of the interviewees mentioned that they keep some aspects of their personal lives (e.g., family, relationship, financial issues) private and untouched while sharing a lot about the rest (e.g., daily routines, whereabouts, product reviews). It is interesting to realize that SMIs seem to have their own definition of “privacy” and set clear boundaries regarding what to share with followers and what not to.

Furthermore, we explore the downsides of being digital labor in the influencer industry. On the one hand, our findings add to the current discussion on digital labor (Scholz, 2013; Neilson, 2018; Archer, 2019; Meisner and Ledbetter, 2022), aesthetic labor (McFarlane and Samsioe, 2020), and sexualized labor (Drenten et al., 2020). On the other hand, these findings extend our understanding regarding the wellbeing of influencers in the digital economy (e.g., Ashman et al., 2018; Levesque et al., 2023). These findings corroborate what Ashman et al. (2018) revealed how factors including the fierce competition in the influencer industry, the creativity demand, and pressure to conform to beauty standard affect YouTubers’ life quality and wellbeing. We further added to this ongoing conversation by uncovering how unfair treatment by brands and followers’ negative comments shape influencers’ wellbeing. For instance, most of the interviewees mentioned the un(der)paid treatments by brands or clients, echoing what (Meisner and Ledbetter, 2022) found regarding creative labor in the process of self-branding. Given the unbalanced power relation and inequality, SMIs, often in the early stage of their career, conducted unpaid or extra work to meet brands’ requirements. Second, SMIs narrate their lives to build personal branding and are thus continually subject to scrutiny. Not surprisingly, a lot of SMIs have to cope with hate and harassment from followers or viewers. Duffy et al. (2022) described the systematic and aggregated critiques as “networked hate and harassment,” which often takes place in specific forums (or “hateblogs”) or other communities. SMIs may not always experience these patterned accusations or attacks, but they constantly are met with some followers’ negative comments or harassment. Last, we also noticed that being an SMI, to a varying extent, has a psychological toll on them. SMIs as cultural tastemakers and content generators, serve as the sources of inspiration for many (Lou, 2022) and are subject to the pressure of being constantly creative and prolific. They have to keep up with the fast-moving industry cycle and produce valuable content and likable personalities to maintain their influence. Sometimes, they have to bear the pressure to just “keep the look” – their optimal self-presentation (weight and look) or lifestyles.

### Practical implications

The current findings also promise meaningful managerial implications to influencers, followers, brands, policy makers, and social media platforms. For influencers to curate intimate and long-term relations with followers, they are advised to provide and create value for followers by creating valuable content and offering giveaways. Influencers who proactively offer emotional support can create a more enhanced bond with followers. Furthermore, influencers can also adopt the interactive and co-creation strategy with followers when creating interactions and content, which also contributes to intimacies between them. Disclosing personal lives seems to be another requisite for influencers to curate personalities and strong ties with followers. For brands that look for influencers with promising self-branding, they can gauge their brand equity by checking whether SMIs follow basic principles like authenticity, topic sensitivity, and fact checking. As these principles have been enumerated by SMIs to be the guiding principles of self-branding. For instance, influencers who always disclose sponsored contents from organic contents can signal higher level of authenticity than those who do not. Influencers who have initiated viral topics or posts tend to be more sensitive in content creation than those who have not. Influencers who always post credible information in both sponsored and organic posts can be trusted more than those who got involved in controversial posts infused with misinformation or fake news. Given the imbalanced power relation between brands/marketers and SMIs, brands or marketers should fairly treat SMIs and avoid abusing them in sponsorship deals or other collaborative work. Additionally, policy makers should implement timely and clear regulations regarding systematic hate and harassment targeted toward SMIs, for instance, social media platforms should assist regulatory bodies in ensuring online safety and facilitating civilization among users.

### Limitations and future research

This study is not without limitations. First, we acknowledge that the generalizability of findings from qualitative in-depth interviews may be limited due to the small and specific sample of influencers. We suggest that future research can employ quantitative methods to validate and expand upon our findings across a broader spectrum of influencer categories and demographics. This approach would enable a more definitive analysis of trends and differences across various influencer groups, providing a more robust foundation for the conclusions drawn. Besides, to build on the insights gained from this study, future research can explore the experiences of SMIs in diverse markets, such as those in Western countries (e.g., Europe and North America), or in emerging markets like China, where the influencer industry is booming. Studying influencers across different cultural settings could provide a broader validation of our findings and offer new perspectives on influencer marketing. Second, this research is centered on the influencer perspective. It is worthwhile examining how brands, clients, and advertisers view the relation and content strategies adopted by SMIs, as well as the downsides of being a creative laborer in the influencer industry. Last, recent research emphasized the roles of social media platform affordances and characteristics in influencer-follower relation building (Lou et al., 2023). Although the current data did not reveal a significant distinction regarding the effectiveness of different content modalities on relationship building, we acknowledge the potential impact of platform features. For example, TikTok and Instagram reels and live interaction, could theoretically offer different dynamics in engaging audiences. We suggest that future research can benefit from explicitly focusing on the platform and content type used by SMIs and examining how these preferences align with their self-branding and relationship-building efficacy. Such studies can provide more nuanced insights into the role of content modality in the effectiveness of influencer marketing strategies.

## Data Availability

The dataset generated for this study is only available on request to the corresponding author.

## Appendix A: Interview guide

1. What do you think about the term ‘influencer’?
2. How would you prefer to be called?
3. How long have you been earning an income through social media?
4. Can you share with me how you entered this field of work?
5. Can you share with me the things you do on your social media platforms?
6. How do you continue to maintain a presence on your social media account(s)?
7. Do you have a particular audience you serve?
8. How would you describe your positioning?
9. Did you set out with this image in mind? (If not, is it something that came to you over time as you spent more time in this field of work?)
10. Why do you think people are attracted to you? What are some of the reasons you can think of?
11. What do you think you are able to provide your followers?
12. How do you think you are different from other ‘influencers’ to your followers?
13. How you think about and view your followers? Could you describe your followers in a few words?
14. How would you describe your relationship with your followers?
15. How emotionally close do you feel towards them? Why do you feel that way?
16. Would you call your followers your friends? Why or why not? How are they different from your friends in real life?
17. Do you trust your followers? Do you trust them more than your friends in real life? Why or why not? In which instance would you trust your followers?
18. Would you depend on your followers the same way you would depend on your friends in real life? Why or why not? In which instance would you do so?
19. What do you think makes a good, worthwhile, satisfying relationship with followers?
20. What do you think is important to your followers? Are there things you feel they expect of you and/or your work?
21. What are some ways you try to build a relationship with your followers?
22. Do you employ certain strategies to build a relationship with your followers? What are they?
23. What are some tools you use to establish a strong relationship with your followers? Do you think they are useful? Why or why not?
24. To what extent do you share your personal life or personal thoughts with your followers?
25. Do you draw a line between your private and public life? How do you draw that line?
26. What are some considerations that come to mind when deciding whether to post something that is deemed to be ‘private’ to you?
27. Are there certain aspects/topics you hold back from sharing? Why do you prevent yourself from sharing about them?
28. What are some considerations you have when deciding whether to agree to a paid contract or deal with a client/brand?
29. When did you accept such an offer? Could you walk me through the process or steps you have to take after agreeing to one?
30. How do you present your information when it comes to doing paid posts? How are they different from non-paid posts with brands/clients?
31. Do you think it (being sponsored) affects how your followers receive the post? How so?
32. Have you experienced a time whereby you did not receive proper or adequate payment? Could you share with me about that experience?
33. How did you negotiate with the client/brand? What was the outcome?
34. What factors do you feel should come into consideration when brands/clients come up with a figure for payment for the work done?
35. Do you feel that brands/clients that you have worked with thus far took these factors into consideration? Why do you think they are important?
36. How do you think clients/brands view social media influencers like yourself?
37. How do you think society perceives you (as an influencer)? How do you think society perceives your work?
38. What do you wish people knew about your work?
39. What are some personal thoughts you have about the work you do?
40. What do you think of the influencer industry?
41. What are some difficulties you face as a result of being a part of this industry?
42. Which aspects of the industry do you like?
43. Which aspects of the industry do you dislike? If so, is there anything you would like to change?
